# Understanding users’ perspectives on mobile apps for anxiety management

**DOI:** 10.3389/fdgth.2022.854263

**Published:** 2022-09-01

**Authors:** Andreas Balaskas, Stephen M. Schueller, Anna L. Cox, Gavin Doherty

**Affiliations:** ^1^ School of Computer Science and Statistics, Trinity College Dublin, Dublin, Ireland; ^2^ Department of Psychological Science, University of California, Irvine, CA, United States; ^3^ Department of Informatics, University of California, Irvine, CA, United States; ^4^ UCL Interaction Centre, University College London, London, United Kingdom

**Keywords:** mental health, mobile apps, mobile interventions, stress, anxiety

## Abstract

Anxiety disorders are the most common type of mental health problem. The potential of apps to improve mental health has led to an increase in the number of anxiety apps available. Even though anxiety apps hold the potential to enhance mental health care for individuals, there is relatively little knowledge concerning users’ perspectives. This mixed-methods study aims to understand the nature of user burden and engagement with mental health apps (MHapps) targeting anxiety management, in order to identify ways to improve the design of these apps. Users’ perspectives on these apps were gathered by analyzing 600 reviews from 5 apps on the app stores (Study 1), and conducting 15 interviews with app users (Study 2). The results shed light on several barriers to adoption and sustained use. Users appreciate apps that offer content variation, customizability, and good interface design, and often requested an enhanced, personalized experience to improve engagement. We propose addressing the specific app quality issues identified through human-centered design, more personalized content delivery, and by improving features for social and therapeutic support.

## 1. Introduction

Mental health disorders are among the leading causes of disability. It is estimated that one third of the population worldwide will be affected by a mental health disorder during their lifetime (1,2). Anxiety disorders are among the most common mental disorders with an estimated 264 million adults experiencing anxiety worldwide (3). Anxiety disorders affect functioning in daily life, cause avoidance behaviors, excessive feelings of worry and fear, and a variety of physical symptoms such as sweating, and increased heart rate (4).

The scale of the problem has spurred the development of an array of smartphone applications for mental health (MHapps), and it is estimated that more than 10.000 apps targeting mental health disorders are available (5). MHapps can be used in conjunction with therapy, as standalone treatments or for use in prevention of mental illness (6). The vast majority of those apps which target anxiety disorders do so by offering self-management techniques and exercises, providing supportive resources, and enabling real-time and asynchronous exchanges with mental health care specialists (7–9).

Despite the proliferation of MHapps, the majority of them fail to gain traction (10) and sustained use is even rarer (10–12). Prior research has shown that mental health apps can be effective for reducing symptoms of anxiety (13,14). However, apps are not used daily even though the number of app installs may seem high (12). Engagement is as an important factor for the effectiveness of digital health apps and prior research has tried to determine the concept of engagement in different contexts (15–18). For example, a recent review attempted to understand engagement by identifying different features of MHapps for anxiety that encourage regular use or make the app content more appealing. Apps were identified by a search of the most prominent mobile app stores (Google Play and Apple store) using anxiety and cognitive behavioural therapy (CBT) related search terms. The results showed that apps integrate a range of functionalities and engagement features, and the provision of these features is highly uneven (19). Engagement in clinical research is defined and associated with objective/behavioral metrics of use of or interactions with a mental health intervention, such as the number of log-ins or time spent using the technology (20,21). Engagement also entails users’ subjective experience which may be a mechanism for change in clinical outcomes (20,22). However, our understanding of users’ perspectives on such apps is limited.

Self-management is a critical component of many mental health interventions, this however places a burden upon the user. To design for engagement, it is crucial to understand users’ reasons for and the barriers they perceive to using these apps. This would provide researchers in digital health with a better understanding of users’ self-management practices with such apps, and of the different design aspects that affect user adoption and sustained use. This understanding will in turn facilitate developers in the development of apps that are usable and more effective.

In this regard, research has mainly focused on analyzing user reviews to understand users’ opinions of mental health apps. Several efforts have been made for specific mental health disorders such as depression (23), bipolar disorder (24), and sleep disturbance (25), for specific types of app such as mood tracking (26,27), or for specific types of psychological treatments such as cognitive behavioral therapy (CBT) (28) and mindfulness (29). Other studies have focused on gaining an understanding of specific target group users, such as exploring adolescents’ perspectives on mental health apps (30), and mood-tracking apps considered useful to young people (31).

A number of studies have explored users’ opinions and expectations for mental health mobile apps in general (32–36). These studies provide knowledge regarding functionality and aspects that affect user experience with these apps. For instance, we know that users find mental health apps more often through social media, personal searches, or word of mouth as opposed to professional sources (36). Users value apps that are easy to use (32,34,36), offer an aesthetically pleasing interface (32,35,36), and low-cost subscriptions (35). Additionally, we know that users prefer apps that allow customization of the interface (32,34,35), offer high quality content, and adaptive functionalities to user needs (32,35). Other features often desired by users include tracking, the provision of reports and insights, ability to share data, and notifications (36). Users engage with CBT apps that offer the ability to monitor and reflect themselves and the ability to provide different interactions for patients and clinicians (37). Other facilitators that influence user engagement are social connectedness facilitated by the intervention, increased insight into health, and a feeling of being in control of one’s own health (38).

Prior research reveals that reasons for disengagement from mental health apps include usability issues, lack of content variety, lack of personalization and customization options, customer support issues, and data privacy issues (32,35,37–39). Another study reported that apps are not sufficiently able to emotionally support users, may distract users from real life, may create misinterpretation about themselves, and may discourage face to face interactions (33).

Previous studies have aimed to better understand the physical and mental health apps people with symptoms of depression and anxiety use and for what purposes (40), and examined features young people like and dislike in smartphone apps for anxiety and depression (41). The results revealed that people use mental health apps specifically for training or habit building purposes (40), and the features liked by young people include autonomy, simplicity, the ability to personalize experiences, and social features that allow connecting with others (41).

Understanding users’ needs and expectations for mobile apps targeting anxiety disorders has received little attention however. Anxiety disorders are among the most common mental health conditions, and coping with anxiety is a common reason people report searching for mental health apps (42). The clinical symptoms of anxiety disorders raise some unique considerations for apps intended to support anxiety self-management. Anxiety disorders are characterized by an intense emotional response to a real, perceived (fear), or future (anxiety) threat and associated behavioral disturbances such as avoidance. These disorders differ from each other, however, in terms of the things that cause fear or anxiety (43). For example, fear of specific objects of situations is characteristic of specific phobias, fear of experiencing a panic attack leads to panic disorder, and fear or anxiety over various events or activities is characteristic of generalized anxiety disorders. The most common treatments for anxiety disorders are those based on cognitive-behavioral therapy (44). CBT techniques include both interventions aimed at increasing one’s ability to cope with anxiety in the moment, such as distress tolerance skills as well as interventions intended to build long-term skills to address anxiety such as exposure therapy or cognitive restructuring (45). Mobile apps that integrate CBT are being some of the most empirically supported apps for anxiety (14). Some of these interventions are well-represented in MHapps for anxiety management, including practice elements such as relaxation, mindfulness, and coping, whereas others such as exposure are rarely used (46). The symptoms of anxiety disorders might also impact one’s motivation and interest in using a MHapp to self-manage these symptoms. Emotional and physical symptoms of anxiety, such as fear, anxiety, increased heart rate, rapid breathing or shortness of breath, and feeling faint, might come on quickly when faced with an object or situation related to one’s disorder. In these cases, a person might be motivated to reduce this distress and use a MHapp to do so. However, even in anxiety disorders these symptoms tend to diminish once that object or situation is gone, which might reduce one’s interest in using an app.

As such, the characteristics of anxiety disorders and the treatments that are effective at addressing them, might guide the features of apps intended to promote anxiety self-management.

Apps are not always optimized to support clinical use and benefit (47). Although some research has examined users’ opinions of mental health apps for specific conditions and target groups, there is a need for a greater understanding of users’ burden and engagement with mobile apps for anxiety disorders.

The aim of this paper is to understand the user burden of, and motivations for, engagement with mobile apps for anxiety management. More specifically, we seek to understand user needs and expectations for the self-management of their health through the use of those mobile apps, which factors facilitate or hinder engagement, and how apps can be improved to better support their mental health. Through this understanding, we seek to improve the design of mobile applications targeted for that purpose.

## 2. Objectives

The studies we report in this paper contribute knowledge regarding user expectations of mental health apps for anxiety, the functionality they see as useful, and the barriers to engagement that they encountered. The main research questions addressed are: What are the views and perspectives of users on the functionality of mobile apps for anxiety? What are the main barriers to engagement? How could the apps be improved to support user engagement? This paper provides an understanding of users’ perspectives on engagement with mobile apps for anxiety management to inform the design of effective applications. Through this understanding, we seek to improve the design of mobile applications developed to support this user group.

We aim to build a holistic understanding by gathering rich data from two methods to counterbalance inherent biases in each method. The data sources we utilize comprise reviews from app stores (Study 1); and interviews with the users of apps designed to support anxiety management (Study 2). Study 1 helps to identify the barriers to engagement, unmet requirements, and reasons for sustained use. Study 2 explores these issues further by investigating positive and negative experiences with apps in-the-wild, understanding user expectations for apps targeting anxiety management, and exploring how apps can be improved to facilitate engagement. These studies aim to analyze user perspectives on the enablers and barriers to their use of such apps rather than to evaluate the quality of individual apps. User reviews allowed the collection of perspectives from a broad range of users, but contain only information that the users consider important and see as appropriate to put forward in app reviews. It is also unclear if users of Study 1 are using the apps for anxiety management purposes only and for how long users are engaged with an app. Therefore, we conducted an interview study to complement observations naturally offered by users in Study 1 by allowing researchers to ask more direct questions about the real-world usage of apps used for anxiety management only. Both studies contribute to a set of design implications and recommendations produced through the synthesis of these complementary perspectives.

## 3. Study 1 - app reviews: understanding the main barriers and facilitators to engagement

### 3.1. Method

**Figure 1:**
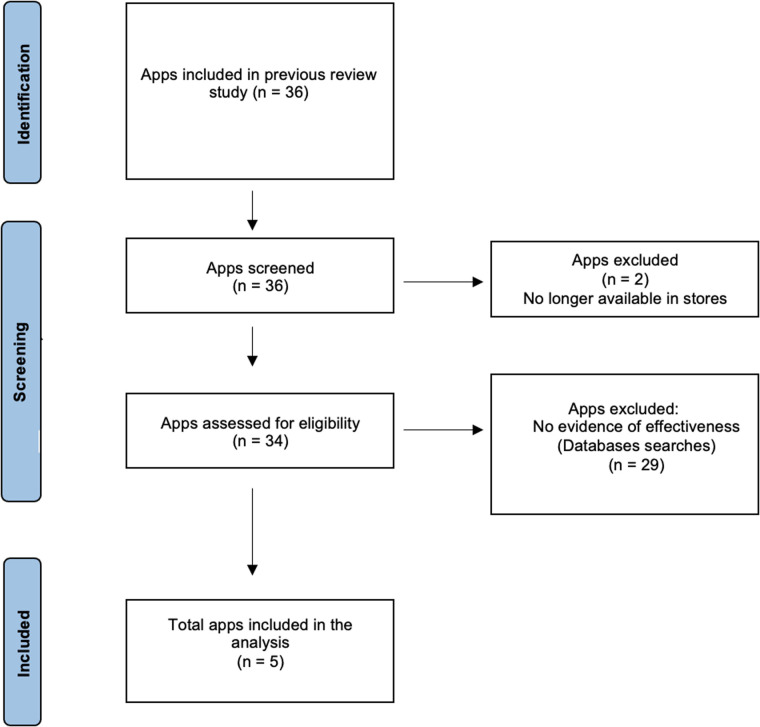
Flow diagram for app eligibility.

Our first study seeks to identify the features considered useful and most important for the users of mobile apps for anxiety, and the barriers preventing sustained use. The set of candidate apps for inclusion was taken from the recent review of anxiety apps mentioned earlier which examined how cognitive behavioral elements are delivered by anxiety apps, and their functionalities to support user engagement and tailoring based on user needs (19). In that study, apps were identified by a search of the Google Play and Apple app stores using the following anxiety and cognitive behavioural therapy (CBT) related search terms: anxiety, stress, worry, phobia, panic, and cognitive behavioural. Apps were included if they belonged in the app store category of Health & Fitness, or Medical, mentioned the use of CBT and anxiety related disorders in the title or store description, were currently available to download, and available in English. Starting from this list of the apps, we further searched for a subset of apps that present some evidence for effectiveness for anxiety management (48–55), with empirical research demonstrating the app resulted in reductions in anxiety symptoms. We used an app rating platform that reviews digital tools (including both apps and web-based programs) for mental health and wellness (https://onemindpsyberguide.org/) and allows consumers and clinicians to identify high-quality apps, including those with demonstrated research evidence. We consulted the apps’ credibility metric which includes a synthesis of the research evidence for that app (56). Additionally, we conducted literature searches to identify any additional published studies demonstrating effectiveness for reduction of anxiety symptoms. This resulted in a total number of five apps for inclusion in our study. The app selection process is illustrated in Figure 1. Details about app features and characteristics are available in the Supplemental Table 1.

The analysis of publicly available app reviews has been successfully used in the past and allows us to gain insights into consumer perspectives on mobile apps, and identify specific aspects of functionality that users value (23,24,26,35). These reviews can be sorted based on rating scores (ranging from 0–5), and based on positive and critical reviews. Android and iOS app reviews were exported in a CSV file by using an online software tool (Appbot.co). We downloaded all the available reviews for each app included in our study. The initial search yielded approximately 140.917 reviews in total, from which we selected a subset of reviews for qualitative coding. There was a diversity in the age and number of reviews among apps and app stores. The goal of analysis of these reviews was not to evaluate individual apps, but rather to identify barriers and enablers to use from a user perspective. Therefore, we selected a random subset of 20 reviews for each possible rating (ranging from 1 to 5) and included reviews that were longer than 20 characters. This resulted in a total of 100 reviews for analysis for each app. Positive reviews were often general in nature and did not provide specific details on which features of the app were valued. For example, R348 “I really think this app helped me with my anxiety and also it helped with my every day mind set five stars I love it.” Therefore we selected a subset of 20 additional reviews per app with a rating of 5 to reach data saturation. The lead author manually assessed all reviews, and conducted a thematic analysis of these reviews, following the framework of Braun and Clarke (57). The framework consists of six steps including becoming familiar with the data, generating initial codes, searching for themes, defining themes, reviewing themes, and writing up the results. We used a single coder approach, where the first author iteratively identified codes from the data, and refined themes throughout the analysis. Coding was done by the first author and regularly discussed with the other authors to allow for better familiarization with the data and reduce potential for bias. The authors then agreed upon a final coding framework to be applied to the remaining data. This iterative process led to codes gradually being merged into broader categories and researchers identifying overarching themes. One researcher reread the entire data set before defining the themes to review the validity of individual themes in relation to the data set.

## 4. Study 1: findings

**Figure 2:**
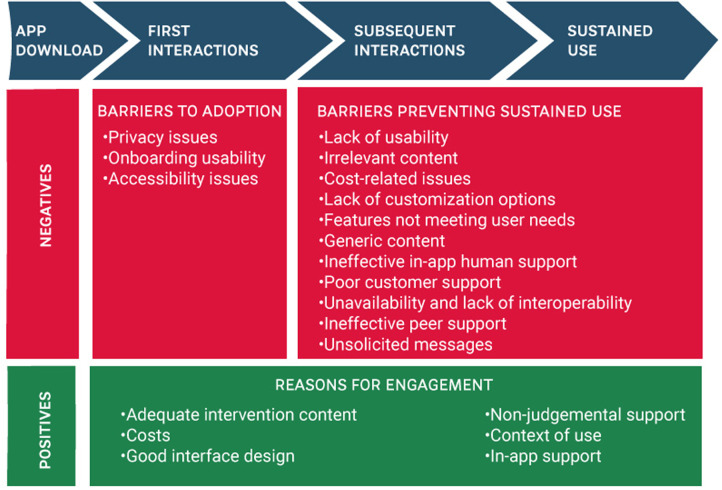
Factors affecting user experience with MHapps.

The 120 reviews from each app resulted in 600 reviews for analysis. We present the results from user reviews according to positive and negative aspects that affect user experience. A few both positive and negative reviews included a contrasting statement, most commonly a request for additional features. We recorded and reported separately the content of reviews that used a contrasting statement. We identified and categorized reviews into barriers to engagement and reasons for engagement. This categorization helped us explore the different factors that affect user adoption and sustained use in different parts of the user journey. Figure 2 shows the factors affecting positively and negatively the user experience with the apps. Additionally, we merged or categorized themes that were closely related and categorized themes based on their relevance. We present the results by referring to reviewers’ comments using a unique number for each identified review. User reviews are included in exactly the same words as were used originally, including any possible spelling and grammar mistakes. Supplemental Table 2 contains more examples for each theme.

### 4.1. Barriers to engagement with apps

The analysis of the reviews revealed several factors negatively affecting the experience of users with the apps. We distinguish the barriers to engagement into two categories: barriers to start using the app and barriers preventing sustained use. We define sustained use as the ability to continue actively using an app for an extended period.

#### 4.1.1. Barriers to adoption

Three factors affect negatively the experience of users as they start using an app: *privacy issues (N=31), onboarding usability (N=13), and accessibility issues (N=6)*.

##### 4.1.1.1. Privacy issues

Users are concerned about their data privacy and security when privacy policies fail to provide clear or concise information on data collection, handling, sharing, storing, and use. “This is a great idea, and I’d love to try it, but. It requires an account, and sharing data with a company. This is the kind of data you should never trust a company with - many will simply sell it on - this is how they make money. Others are more trust worthy but get bought or hacked. Deleting it until there’s an option to share no data”[R53]

##### 4.1.1.2. Onboarding usability

Another barrier to adoption is the presence of bugs preventing users from creating an account or resetting their password, for example “The app doesn’t let me log in, even resetting the password doesn’t work. This made a bad day worse. Have to find another app” [R311].

##### 4.1.1.3. Accessibility issues

Barriers to accessibility make apps difficult or impossible for some people with disabilities to use, for example “Not accessible with voiceover. I am almost blind and I can’t work with this app. Also, with what little vision I do have, there is no dark mode available and it look like I am looking at a blank screen with circles of color. Please make this accessible for blind and visually impaired users.” [R351]. Additionally, the specified target audience of the apps was misleading preventing the use for specific groups of people. For example, “It says in the App Store 4+ yet when you open the app the youngest age is 18. What about younger people? What do we do?” [R477].

#### 4.1.2. Barriers preventing sustained use

Several factors impact a user’s ability or motivation to persist with an app over time. These factors might not impact an initial decision to use an app but would have an impact on sustained use. The factors negatively affecting the user experience while using an app are *lack of usability (N=87), irrelevant content (N=85), cost-related issues (N=67), lack of customization options (N=66), features not meeting user needs (N=51), generic content (N=26), ineffective in-app human support (N=18), poor customer support (N=15), unavailability and lack of interoperability (N=10), ineffective peer support (N=5), unsolicited messages (N=2)*.

##### 4.1.2.1. Lack of Usability

Some factors affect the degree to which an app allows users to perform the tasks safely, effectively, and efficiently while enjoying the experience (58). Usability factors that negatively affect the experience of interacting with an app are related to issues with the design of the interface. User experience is disrupted when navigation is not taken into consideration when designing apps preventing access to app features or redirecting to the wrong location. For example, “It’s incredibly clunky to navigate through this app and tell what’s what and where it is” [R201], and “Going back to home removes the previous conversation it’s annoying as hell…”[R110]

Other users were frustrated by the presence of many elements on one page or the lack of guidance during use. For example, “The app is quite chaotic. There are no guidance to read before I get started or explanation as to why I’m doing all this” [R263].

Sustained use is affected by functionality issues such as bugs causing app crashes, update issues, and broken functionality of app features. Lastly, users discussed an app’s incapability to respond quickly to their needs due to slow response times, for example: “Nice app to distract brain on rainy day. Please check its performance, its been painfully slow lately. Checked on multiple phones I have” [R434].

##### 4.1.2.2. Irrelevant content

Apps and mainly chatbot apps provided users with responses that were not relevant to their unique inputs. For instance, “I do like this app but at times it’s responses doesn’t take into account what you’ve actually said. When it does it is pretty good” [R76]. Finally, a few users reported low-production value content. For instance, “the new meditations are the same thing except read in a scratchier voice and sped up 1.25×. It’s hard to meditate when you get a new prompt every 15 seconds…..” [R304]. Users were dissatisfied with different elements used to deliver intervention content such as excessive use of emojis, and the language used for that purpose. For example, “I would rate this app before four of five although it has appropriate emotional language that younger youth may understand I think older adolescents may not identified with those presented and may want to elaborate their feelings…” [R371], and “The 5 minute ‘float through the cloud’ audio uses words like ‘untroubled’ and ‘not worried’ (rather than ‘serene’ or ‘peaceful’) so the sleepy listener is reminded of worry and trouble. A CBT practitioner would know this…” [R112].

##### 4.1.2.3. Cost-related issues

Some reviewers discussed barriers associated with costs that negatively affect their experience with the apps. Users feel that apps offer costly in-app purchases and subscriptions, and complained about hidden costs when purchasing a subscription. Additionally, existing app users are affected by new payment plans that block content after an update. Users mentioned that apps provide limited content in the free version, complained about unexpected payment patterns during app use, and felt that premium sale is forced. For example, “.. However I am pissed that the ‘free’ tracks are peppered with premium content that is locked. I can’t even have a decent test run of the content” [R416].

In addition, consistent bugs have an adverse effect on user’s willingness to purchase a subscription plan, for example “I’m not sure I’m gonna pay for the full version of even the free stuff doesn’t work right” [R221]. Lastly, an unexpected invoicing process disengaged some users from interacting with an app. For example, “Don’t bother. They obsessively bill your bank account way before it’s due and have poor communication with billing on the app.” [R221].

##### 4.1.2.4. Lack of customisation options

Several users were unable to customize the interface of app features and requested more options to respond when interacting with an app and options to recall a decision during an interaction. For example, “Needs a ‘maybe’ button between the ‘yes’ and ‘no’ and a way to say, ‘You’re on the wrong track,’ before it decides it knows all your usual problems and keeps assuming them over and over with no way to remediate” [R64]. Additionally, users valued the possibility to use the app in different contexts and prevent screen lock while practicing specific strategies such as breathing exercises.

Users want the ability to track their mood more often and be offered more self-monitoring options. Similarly, users prefer enriched reflection on the collected data they provide through the apps. For example, “I’ve been using this app everyday for about two months. In theory, the bot should be learning about me or identifying trends, but that never happens. The ‘mood over time’ screen will only show your mood over the last 5 days or so, and there’s nonway to look back further in time. There is no feedback for CBT” [R14].

Users also requested localization of app content and adaptation of app features in their area of residence. For example, “What about adding Italian? Meditating in a foreign language is not relaxing” [R273], and “It seems that because coaching is not available in my area a large part of the application is not relevant” [R282].

Users requested options to customize reminder timing but there were mixed views about the number of notifications they would like to receive. For example, “I also wish it had a bit of a stronger presence (checking in on you when you haven’t used it for a while, for example)” [R26], but R54 stated “But what really bothered me about the app was the first reminder I got when I didn’t use the app a second day in a row because it sucked was definitely guilt inducing.”

##### 4.1.2.5. Features not meeting user needs

Several users mentioned that the content did not address their needs or had opposite effects on their mental health. Users sometimes felt that the interaction with the app did not provide any benefits to them, and the content was not helpful. Additionally, lack of content variation and updates on content delivery can have an adverse effect on user experience. For instance, “Also the app didn’t give me a lot options to work out MY stress. I wouldn’t recommend this app” [R463].

Apps are not designed to assist with specific conditions such as addressing trauma and/or abuse. Additionally, app content did not adapt to unexpected situations such as the pandemic. As R473 stated, “I used it for 6 months but it doesn’t account for people wanting to take a break on the weekends. It as heck was not helpful during the COVID. A lot of the stuff was unrealistic during a pandemic if you were stuck at home and could not leave” [R473].

##### 4.1.2.6. Generic content

Several users discussed the issue of receiving repetitive content when interacting with a chatbot app. For example, “Repetitive keyword-based therabot. I liked the idea of having someone to chat with when I needed it, but really all this bot does is ask ‘How does this make you feel?’ and give canned responses to keywords. If you need a therapy app that walks you through, this might help you. But if you’re looking for conversation to help you feel less alone, this won’t help” [R113].

##### 4.1.2.7. Ineffective in-app human support

Apps can support users through their journey by providing access to counseling sessions through the app. User reviews showed that in-app support can have a negative impact on their experience for a variety of reasons. Users expressed dissatisfaction with the therapist support provided through the apps, feeling that they accrued no value through the sessions, or that therapists were judgemental. Additionally, users discussed the lack of therapeutic alliance and in-person contact with a therapist due to the nature of a text-based interaction. For example, “I don’t think text based therapy is working out for me. I just don’t think it is personal or effective enough for me… I had a therapist I was talking to called ⟨name⟩ and now suddenly I get a new one for my next session. I would have loved to continue and build the relationship with one therapist” [R152]. Moreover, the text-based nature of counseling sessions results in slow response times from the therapist. For example, “BUT I don’t recommend THERAPISTS. In 30 minutes, around 10 are spent waiting for them to read and answer, feels like they have someone else talking to…” [R132]. Lastly, users also disengage from receiving therapist support through the apps when different time zones affect a therapist’s availability.

##### 4.1.2.8. Poor customer support

Users disengage with an app when assistance from an agent or support team is ineffective or not provided to them. For example, “I tried to change therapists but there is no way to contact this company” [R157], and “I try to contact them but very poor customers service…. Be aware…” [R358].

##### 4.1.2.9. Unavailability and lack of interoperability

Users referred to the inability to use an app in different contexts or during a crisis when an internet connection is not available, “It was good the first couple of days. But there is no offline mode so if your in crisis you can’t use the app…”[R15]. Additionally, apps are not designed to work across multiple devices and do not support data transfer across devices preventing sustained app use. For example, R277 stated, “…Plus, it’s one more device. So, I thought I’d simplify things: recycle the iPod and use the app on my iPad. Except that the app doesn’t rotate to landscape, which is how practically everyone uses iPads. It only displays in portrait. If an app doesn’t display on an iPad the way iPads are used, then it’s not an iPad app, just an iPhone/iPod app.”

##### 4.1.2.10. Ineffective peer support

Some apps incorporated peer support by integrating discussion and chat groups for a variety of topics related to mental health. Such support may be ineffective for specific target groups, for example, “The app have several negatives. I’m in my late 40’s. To me, it seems everyone on the app are in their 20’s. The communities are gear towards college students with jobs” [R263]. Additionally, one user mentioned the lack of a feature that allows them to block people on the discussion groups and another one requested a function that allows them to show empathy to other user posts. For instance, “Community is okay but be forewarned there’s no way to block or mute someone. I had someone commenting triggering stuff on all my posts for a while and had no way to avoid them” [R212], and “The only real issue I have with it is that people don’t get to say what the think in terms of I agree or disagree on posts like if someone says they are struggling and you like it to say you agree then it could be taken as you liked that they were struggling” [R284].

##### 4.1.2.11. Unsolicited messages

A few users received unwanted messages when stopping use of the app, for example “This is really annoying. After I deleted the app, it kept sending me messages like, ‘You will feel a million times better after…’ I hate it…” [R463].

### 4.2. Reasons for engaging with the mobile apps

Positive reviews revealed reasons provoking sustained use with the apps. The factors positively affecting the user experience while using an app include *adequate intervention content (N=353), cost-related factors (N=47), good interface design (N=42), non-judgemental support (N=24), context of use (N=20), in-app support (N=14)*. The review analysis showed that adequate intervention content was significantly the most common reason for engaging with the apps.

#### 4.2.1. Adequate intervention content

Users mentioned improvement in their mental health state and indicated that the app content was helpful. For example, R576 stated, “I suffer from general and social anxiety and this app has been so much help!!! If you suffer from anxiety a lot or every so often I definitely recommend this app. It calmed me down so quickly!!.” This response also suggests that the app is being used in-the moment during episodes of high anxiety.

Users appreciated high-quality content and content variation in the apps. For example, “Good app. Lots of features to teach you about anxiety and different types, as well as features to help you. I find myself most frequently using the thought journal, but I’ve used most of the features” [R337]. Additionally, users value apps that are accessible at any time, and provide evidence-based content.

Apps offered users the opportunity to learn new tools, and provided insights about themselves. For example, R82 stated, “I find this App helpful to learn tools and definitions associated with my anxiety, and the daily check-in is reassuring.” Finally, users value content customised to their needs. As R293 stated, “The information has been important and relevant to what I am going through since it is customized to me.”

#### 4.2.2. Costs

Users value apps that are free or at an affordable price, provide adequate content in the free version, and do not restrict access to content behind a paywall. In addition, users take advantage of alternative payment options such as fees covered by their insurance. For example, “This app was made available through work benefits to support us through this pandemic. This was honestly something I needed but did not know how to get” [R553].

#### 4.2.3. Good interface design

Users often valued aspects of the interface design such as an app’s user-friendliness, simplicity, and aesthetically pleasing interface. For example, “I really really like this app. It’s simple, clean, visually good, complete and very helpful” [R332]

#### 4.2.4. Non-judgemental support

Users feel that apps are non-judgemental and substitute of other forms of interaction. For example, “Things have been tough lately at home and I didn’t want to open to family. With this app I thankfully have someone to talk to and help me out” [R534], and “I have always been someone who struggles to open up and tell people about my problems, but this app makes that so much easier and is a good place to vent without fear of being judged…” [R194].

#### 4.2.5. Context of use

The reviews revealed different contexts under which apps are used in daily life. Apps are used as an alternative to therapy. For example, R597 stated, “I don’t have access to mental health treatment/therapy but this is a good alternative for now. It helps me practice positive thinking and being aware of how my thoughts affect my wellbeing” [R597]. Other users interact with the app while on the waitlist to receive therapy; “This app has really helped me while waiting for my first therapy session” [R506].

Apps are used in-between therapy sessions either by users integrating apps into their therapy sessions or by therapists who recommend apps to their clients. For example, “As a therapist I have referred many of my clients to this app. It really is very helpful” [R372].

#### 4.2.6. In-app support

Users can be satisfied with the therapist support provided through the app. For example, R554 stated, “The coaches are also really well-trained and thoughtful with their responses.”

Users value the variety of groups offered through social support features, and the opportunity to share their experiences with other people. For example, R246 stated, “…and I really enjoy being able to chat in the community section and choose specific topics has needed. 10/10 would recommend,” and “The community chats are also really nice and make me feel like I’m no o the only one struggling. It really is a great app” [R235].

### 4.3. Limitations of study 1

Prior research has examined the reasons for disengagement from mental health apps (32,35). The results from this study distinguish the reasons that negatively affect engagement into barriers affecting user adoption and barriers preventing sustained use. The app reviews analysed in this study allowed identification of several issues that affect user adoption and prevent sustained use. However, such reviews have the limitation of including only those details the users saw as relevant and were willing to disclose in the form of a public review. While natural and unprompted, user reviews were brief and did not provide in-depth information. In addition, we were unable to determine the factors that influence how and why people choose to use apps to support anxiety management. This form of study does not support us in probing specific issues such as how apps are used in real-world settings, for how long, nor if users are using/have used such apps for anxiety management purposes only.

Therefore, we conducted a second study to explore the in-the-wild experiences of users with anxiety management apps to understand in more depth the motives for use, the challenges they encounter with such apps, and suggestions for improvement. The results from the interview study captured information related to app selection, users’ motives, and expectations for such apps; information related to the real-world selection and use of such apps which could not be captured from the analysis of user reviews. Thus, the interview results allowed us to further understand the user experiences with the apps and to elicit suggestions for improvement. Combining insights from these two studies would support the analysis of complementary user perspectives on the enablers and barriers to engagement with apps for anxiety management.

## 5. Study 2-interviews: exploring peoples’ experiences with mobile apps for anxiety

### 5.1. Method

In order to address the limitations of study 1, our second study further investigates users’ perspectives on mobile apps for anxiety. Specifically, we investigated how and why people use apps to support their mental health in relation to anxiety management, challenges users encounter, and suggestions for improvement. To explore these issues we collected qualitative data using semi-structured interviews. Topics discussed during the interviews comprised positive and negative experiences with the apps, features users liked/disliked, desired features, and suggestions for improvement. We conducted interviews that lasted about 30 minutes and were recorded and transcribed by one author. The study was approved by the relevant institutional research ethics committee.

We recruited 15 participants from June to August 2021 via social media, and the researchers’ personal and professional networks. Participants were required to be at least 18 years old, be proficient in English, and have used or are currently using a mobile app to support their mental health in relation to anxiety management. Social media posts and emails listed these eligibility criteria, the length of study participation, and the study compensation. All participants received a 20€ voucher upon completion of the semi-structured interview. Several procedures were in place in case participants experienced discomfort during the interview^1^. The interviewer transcribed all recordings. Transcripts were then coded and analyzed using a general inductive approach (59). The primary purpose of the inductive approach is to allow research findings to be generated from the frequent, dominant, or significant themes inherent in raw data. This is a more descriptive approach that seeks to understand the people’s perspectives. It’s flexibility allows the development of categories and topic summaries, as well as themes. It suits the analysis of interview data as it acknowledges the more targeted nature of the interviews, and the specific questions posed. One author engaged in close reading of responses and developed initial categories through inductive coding. The author then grouped categories with similar meanings into broader categories to develop the themes. The categorization of codes and themes were then discussed with the last author.

Table 1 presents participant demographics. The majority of participants were women, consistent with data suggesting that women are more likely to use mental health apps (60). Our sample had a range of ages and was highly educated with all participants indicating familiarity with the use of technology in daily life. We present the results by referring to participants’ comments using a unique number for each of them.

**Table 1:** Demographic characteristics of interview participants.

Attribute	Range	Sample size
Gender	Female	11
	Male	4
	Non-binary/ third gender	0
Age	18–24	2
	25–34	6
	35–44	5
	45–54	2
Education level	High school degree or equivalent (e.g. GED)	1
	Some college, no degree	1
	Bachelor’s degree (e.g. BA, BS)	5
	Master’s degree (e.g. MA, MS, MEd)	4
	Doctorate or professional degree (e.g. MD, DDS, PhD)	4
Experience with technology	Fundamental awareness (basic experience)	0
	Novice (some limited experience)	0
	Intermediate (experience of using tech in practice)	7
	Advanced (experience using tech in complex projects)	5
	Expert (others come to you to ask about your experience)	3

## 6. Study 2 findings

Several broad themes emerged from the interviews regarding reasons for using mental health apps for anxiety, positive experiences, barriers to engagement, and suggestions for improvement.

### 6.1. App use

Participants reported using a diverse set of apps to support their mental health in relation to anxiety management, and 45% of them are using more than one app for that purpose (See Table 2). Participants use multiple apps to access different features on each of them, or use apps with the same functionality but with specific preferences for content delivery among them. Participants support their anxiety by using either one specific app function (e.g. to meditate) or with the use of a variety of features offered through the apps. Almost half of them have used or are currently using the same apps to meditate (i.e Calm and Headspace). Interestingly, two participants use other health apps to support their mental health. One of them uses a health app to check her sleep quality and a mental health app to meditate. The other participant has stopped using mental health apps and uses only health apps to monitor his sleep patterns and to meditate. Most of the participants are currently using the apps even though a few of them reported noncontinuous periods of use based on life events or improvements in their mental state. Table 2 presents participant experiences with mental health apps.

**Table 2:** Characteristics of mental health resources.

Participant	Main sources/App/s Name/s	Other
P1	Calm harm, Garmin	SoundCloud
P2	Fitbit, Headspace	Youtube meditation videos
P3	Wysa	
P4	Insight timer	
P5	Calm, Headspace	
P6	Calm, Balance	
P7	Calm, Sanvello, Headspace	
P8	Medito	
P9	Woebot, Insight timer	
P10	Powerflower	homework between therapy sessions
P11	Calm, Insight timer	
P12	Calm	Relaxed melodies app
P13	What’s up?	
P14	Welltrack	meditation podcast
P15	Calm	
Length of app use		
>1 month	1	
>2 months	2	
>4 months	1	
>6 months	2	
>1 year	7	
>2 years	1	
Timing of app use		
Current users	11	
Past users	4	

The majority of participants are currently using mobile apps to support their anxiety. Six participants use the app because they noticed an improvement in their mental state. Another three still use the apps because their use has been integrated into their daily life. For example, P13 stated, “I still use the app because it has become a habit to me because even maybe when I am unwell I still need to track what I’m doing.” Other reasons for using the apps include awareness of one’s self, low cost, dedication to subscriptions, and the willingness for continued practice to support future anxiety events. One participant uses the app in addition to having paid the yearly subscription because of shared experiences with friends. As P11 stated, “So yeah, I think I will continue using it. And also a couple of my friends use it and they’ll sort of say their message me. And they say, have you seen today’s daily calm? It will really speak through some of the things you’re going through. And so you can kind of and I’ll say have you heard this bedtime story? So because quite a few people are using it, you can have kind of shared experiences.”

Participants stopped using mental health apps because of high costs or based on improvement in their mental health. For example, P3 stated, “I felt like I have gone to the stage that I feel I did not need to use the app. I am on a stage where I am quite busy and my mental health has been ok. I did not feel I need to use the app.” P14 stopped using an app when patterns on the data and treatment elements became non-useful to her, “And then over time, I used it less and less and less. I think basically I wasn’t really seeing any pattern that I wasn’t necessarily picking up anything that was useful. And then I say I got all the tips and things I was just doing the ones that were useful anyway, didn’t need the app to keep doing that.”

### 6.2. Motivations and expectations for mental health mobile apps for anxiety management

Participants discovered apps through recommendations from their social network, through social media, or through relevant institutions. Eight participants received recommendations from other people, 3 participants chose apps based on advertisements in their social media, 3 based on recommendations from participants’ relevant institutions, and 1 based on a search in the NHS digital library. These recommendations included personal and professional sources such as friends, family members, and therapists. For example, P11 stated, “..and then my mum has been using ‘Calm’ and recommended it and I got a free trial and then I accidentally forgot to cancel so I signed for a year so I thought I will keep using it and then ‘Insight Timer’ a colleague recommended it to me and said it is free, this is how you use it..”

Similarly, one of the participants started using an app based on a recommendation from a therapist, P10 “This one I have been using it since February because we had a trauma-focused yoga instructor doing sessions with us and something that he suggested with some of the facilitators for the rest of the therapy sessions, and I have been trying to use it since then.”

Three participants started using an app based on recommendations from relevant institutions. As P7 stated, “Headspace I could get for free through my university and Calm I could get for free through my health insurance, and that kind of encouraged me to really use it in a daily basis.”

Participants’ motives for starting to use a mobile app to support their mental health in relation to anxiety management include stressful life events or recommendations from other people. Six participants started to use an app to improve their mental state in relation to anxiety management. For example, P2 stated, “The main purpose is to calm down and to stop the flow of thoughts and be able to focus because it is impossible to focus when you are super anxious. For me the main goal was to reduce anxiety, that was the purpose.” Five participants reported that they started using an app without having any expectations in particular. For example, P15 stated, “So initially, when I got it, I got it because a friend was using it and she had, like, a free code so you can sign up and use it. And she said it was really, really good. So I got it and I wasn’t really expecting anything from it. And then I started using it and in particular, using it if I was feeling really stressed or anxious, you know? And actually, it has probably the most, it helped the most out of everything I’ve done tried, like, anxiety and stress. So it’s been much more useful to me than I ever thought it could be.” P9 started using an app to check if there any similarities with CBT therapy practices, “I knew it was a chat-based bot and I knew it was CBT and I had CBT, I have done mindfulness based cognitive therapy course in the past so I assume that it was going be some similarities.”

Another participant started using an app in order to integrate a regular routine by practicing meditation daily. Similarly, another participant used two apps, one to manage anxiety symptoms, and another for long-term support of meditation practice.

### 6.3. Positive and negative experiences with mental health mobile apps for anxiety

**Figure 3:**
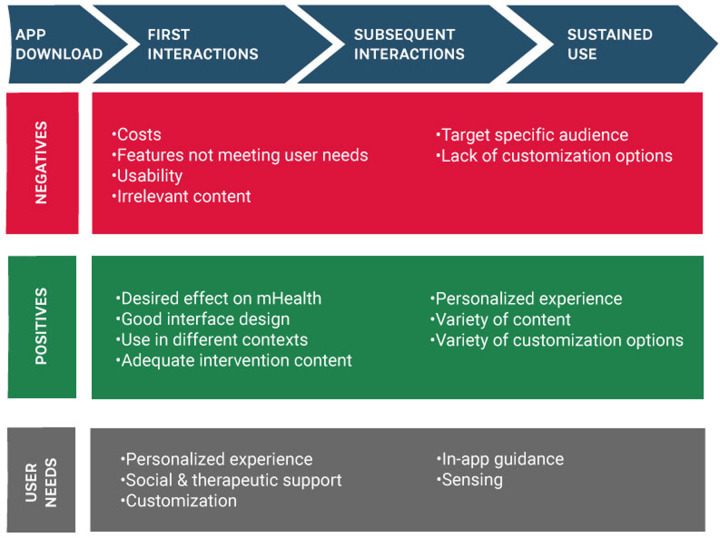
Positive and negative experiences with MHapps.

We detail participants’ responses according to positive and negative aspects of the apps for self-management of anxiety and stress symptoms. Figure 3 summarizes participants’ positive and negative experiences with the apps.

#### 6.3.1. Positive experiences

Interview results revealed reasons affecting their experience positively with the mental health apps similar to the results from the review analysis. Eight participants reported that some in-app features provided benefits to them in terms of addressing anxiety-related symptoms: “So the positive aspect would be that it’s just really helped me, you know, it’s really helped manage my emotions” (P15). Many participants reported satisfaction with the content of the apps and five valued content variety. For example, P4 stated, “I have a feeling with this app, it was been useful in the kind of variety of content so in comparison to other apps which are just like this is what you are getting and that’s it. There is a wide variety of approaches, of topics, of formats on this app. I quite like that because it means I can pick and choose what I feel I need at a particular point I come to.” Other positive aspects include the ability to customize app content and use an app in a time-efficient manner. For example, P11 stated, “And daily calm, because it’s only ten minutes. Yeah, that’s feasible because, you know, I’m so busy, I don’t have time for yoga and meditation. And then you think you’ve got ten minutes. You need to have a screen break from kind of sitting at the computer, so just do it. And so I find that really useful.”

Two participants valued opportunities for low-effort interaction with the apps, for example receiving audio instructions for guided meditation without the need to hold the phone or view the screen, and the ability to use it in different contexts: “And it also supports different conditions, different modalities for instance I am walking in the forest, I am sitting at home doing some mediation or laying in my bed before going to sleep and there is support for that as well so the good thing about it for me is that it supports different conditions during the day” (P2).

Other aspects appreciated by a lot of participants are the aesthetics and the ease of use of the apps. For example, P3 stated, “The Wysa app. I think it is ok, I have good experience with it, it is quite easy to use and seems like the app gives good advice, good personalised tips to you. And it has a nice layout.”

Five participants valued the opportunity for personalised experiences by receiving suggestions based on previous searches or based on self-monitoring data. In addition, one of the participants valued the ability to personalise the experience by selecting activities to be integrated into the algorithm. As P10 stated, “And I also like that you can select your favorite activities, if you found them particularly helpful, it lets you go through the settings and let you know which ones are repeatable, not all of them are but you can have a scroll and tick the ones that you like and works for you and then it will integrate that back to the algorithm which I found I could not do on some of the meditation apps.”

Other positive aspects based on participants’ experiences with the apps include the ability to use an app offline, at any time, and receive encouraging feedback. Three participants appreciated the potential of optional and free app use when interacting with prompts or app content. P4 stated, “It allows you to do what you want without keep asking you to try new stuff or to subscribe to the premium package.” and P10 “I also find that in some of the apps the notifications are a bit of a passive-aggressive, it’s like oh you haven’t done this today and then I think that this is quite passive-aggressive. This one would be like do you want to work on x,y,z today? It feels quite kind. Sometimes I am too sad to do it but today I will look at this.”

P3 reflected on how the experience of self-monitoring through the app provides a space for self-expression “I use the chatbot function, I liked saying how I am feeling kind of having a diary and you do get a response back so it is just like expressing myself because there are not really many places where I can do that. That is the main function I really like.”

#### 6.3.2. Negative experiences

The interviews revealed that the same factors affecting participants’ experiences positively can have an opposite impact if not addressed. These factors include cost-related issues such as perceived high costs, limited free content, and pushed premium content. Other factors include in-app features not providing any benefits in terms of addressing anxiety-related symptoms or the inability to use the app in certain contexts. For example, P2 stated, “For example, if they say sit in the room alone, but maybe I do not have a room where I can stay alone, our previous apartment was really small and it was not feasible you know. So they are restricting you to use the app in certain conditions that you may not be able to create. That is a negative feature. Or maybe it says close your eyes and breathe but you may cannot close your eyes because you are maybe outdoors or you are walking and that is the only time you are alone and it’s not feasible and immediately you are like excluded from this intervention.”

Other factors negatively affecting a user’s experience with the apps are unexpected interactions such as pop-up messages or unexpected sounds during app use, app freezes, and the long duration of some treatment elements. For example, P7 reported, “I never really fully tried them but there are some recordings that are 45 minutes. As soon as I see that I just do not want to do it. I think it would be easier for me to do it if they were broken it up into like 10 minute sessions, so for example they also have courses that I guess are a couple of hours long but they break them down in like videos or like 10 minutes and that’s much easier for me.”

Two participants who used apps with a chatbot as their main functionality reported that factors affecting negatively their experience included misinterpretation of inputs and limited AI responses. Additionally, the language used makes chatbots feel less human-like. As P9 stated, “the AI chatbot is limited, it does try to be funny but it’s not like that funny. I know it is not a real person so I feel that I am talking to an app basically so there is definitely benefit to it but it does not have that humanistic touch which is important I think as well.”

Comments from several participants suggested that they may disengage from apps that provide non-personalized content and limited customization options. For example, two participants reported that the apps target specific demographic audiences which do not suit participants’ interests. For example, P4 stated, “I do not like the landing page when you open the app there is a lot of new content but it is very focused on an American audience I think so you get notifications about things that are happening in the middle of the night but they are for an American audience, although you can access in different languages and there are practitioners from all over the world, I think is still very much focused in North America so all your notifications and stuff are weird and may not accessible in Europe. That is annoying, they just clutter up your landing page with content that is not relevant and there is no option to switch that off.”

P7 reported mixed feelings about the need to interact with the app using the mobile phone, “The thing I love about it being an app is also what I hate so when I want to fall asleep the last thing I want to grab is my phone” Finally, P1 discussed the lack of engagement with all the mental health apps he used to support his anxiety, “The lack of feeling really connected and enthralled by the app such that I want to keep using it, whether it’s daily, weekly, monthly over a long period of time, and the fact that I haven’t got one or two apps that I really call my friends, as it were. That’s the negative experience because the fact that I have an interest in wanting to maybe find an app like that is is a good sign from a technology point of view. But the fact that the app doesn’t match that would be the negative.”

### 6.4. Unmet needs and suggestions for improvement

All participants provided suggestions to improve the experience with the apps to support better their mental state in relation to anxiety management.

#### 6.4.1. Personalization of intervention content

Eight participants requested a more personalized experience while interacting with an app. Such experience would provide them some sort of variation in the suggestive features received based on an app’s history data, or based on specific parameters such as self-monitoring data or other kinds of data. For example, P12 stated, “And don’t keep suggesting me stuff I don’t like and also try and understand me. I’ve never answered any questions about how I feel and what would help me. So it’s really just feeling like there was some kind of more personalized experience and that it learnt from my interaction with it, and I don’t think it’s doing that at the moment. I think it’s just pumping out the same odd stuff that it pumps out to everyone.” Two of the participants discussed receiving content based on specific needs, “Maybe. I guess for me, it’s kind of more focused around particular issues. So if there’s because I’ve got obsessive-compulsive disorder, if I could find content kind of specifically related to that” (P11).

#### 6.4.2. Improved social and therapeutic support

Many participants requested the ability to integrate social features in the apps such as to provide the opportunity to contact a therapist, connect with other people, or download and share progress with a therapist. As P9 stated, “I can’t think of anything to completely improve it other than having an actual coach, like an actual text-based coach instead of the chatbot which might be more effective,” and P13 stated, “So what will make it more engaging is maybe if there will be a chat there where people, maybe it will track you then put you to a relevant group there with other people then maybe you can chat together like an online community to assist each other.”

#### 6.4.3. Customization of input and output modalities

Several participants appreciated opportunities to customize different app features and alter the delivery format of treatment elements. For example, P13 requested, “Okay, I will suggest like the application does have something like you’re able to talk to. Like it should have maybe something like a microphone, you click it. Then maybe talk or your feeling how your day is being,” and P6 “I think maybe having a few variants of voices because you only get like two people a male and a female voice which is fine but some days I do not know I feel I just listened to them so often so you feel you need some variety so having that would be good.”

#### 6.4.4. More detailed in-app guidance

Four participants reported that apps need to provide more guidance on how to use them and explain the benefits of different treatment elements. For example, P10 stated, “I wonder after you completed the activity or whatever the task it would be, it would suggest, it would explain like why is this helpful, why is this beneficial when you may want to use this in the wild. Like this helps you identify the emotion you are feeling and that will help you stay more connected or this helps get your heart rate up which is good for your well-being and fitness in general, this relates to this particular CBT skill.”

#### 6.4.5. Making use of sensor data

Interestingly, three participants wanted to integrate other sources of data such as physiological data into the apps. For example, P7 stated, “I do not know if this could work well in practice but maybe linking it more with physiological data so people can measure heart rates or approximate stress level. If you could measure in a way when people maybe are more stressed or anxious and advice appropriate exercises,” and P2 “Add physiological data, for example, heart rate or some sort of physiological response maybe being able to link it with your smartwatch or some sort of fitness bracelet without really creating their own fitness bracelet but maybe using the technology that you already have is an additional option that could be interesting.”

## 7. Discussion

### 7.1. Principal results

We have presented two studies that investigated factors affecting user burden and engagement with MHapps for anxiety management: in study 1 we analyzed user reviews from app stores to examine factors affecting positively and negatively the user experience with the apps; in study 2, we conducted interviews with users of such apps to further investigate user expectations and motivations, barriers to engagement within the context of use, and unmet needs. These studies provide complementary perspectives, allowing us to build our understanding of reasons for app selection, user motives and experiences with anxiety management mobile apps, and barriers and enablers for engagement with such apps. Our results show that apps are used in a wide range of contexts to cover diverse user needs and expectations. People find anxiety apps through recommendations or search in social media in line with the results from a previous study (40). A good proportion of the participants use more than one app for anxiety management. Almost half of the participants use the same specific apps to meditate (i.e. Calm and Headspace), in line with the results from previous work showing that these apps are among the most used apps for anxiety management (10). People with common MH problems use mental health apps to relax, track their moods, practice mindfulness, self-care, or build healthy habits. They value the ability to self-reflect on the collected data, learn and practice new skills, and progress with their health (61) in line with the results of our study focusing on users of anxiety apps. Users may use multiple apps and other kinds of resources to support their mental wellbeing in relation to anxiety management; they may also have periods of noncontinuous use based on life events and changes or improvement in their mental health. The results indicate that temporary improvement in anxiety symptoms impacts a person’s willingness to engage with an app.

Anxiety causes physical responses to stressful situations, and the nature of anxiety symptoms is linked to deficits in multiple domains of functioning such as sleep problems (62). Therefore, a few participants use health apps to capture patterns in their sleep quality or other kinds of physiological data.

Synthesizing across these two studies identified consistent findings despite data collected in different formats. The user reviews analysis identified the barriers to engagement into two categories: barriers to starting using the app, such as *accessibility issues, usability, and privacy issues*, and barriers preventing sustained use including *lack of usability, costs, unsolicited messages, ineffective in-app support, poor customer support, irrelevant or repetitive content, features not meeting user needs, and lack of customization options.* The user review analysis showed that the most prominent barrier to engagement is the presence of usability issues. Other prominent barriers that affect long term app use include cost-related issues, low-production value content irrelevant to users’ unique inputs and needs, and a lack of customization options. These barriers to engagement are in line with the results from the interview study focused explicitly on users of anxiety management apps and revealed factors affecting user engagement from a diverse set of apps.

Conversely, some of these features (*usability, costs, in-app support, customization options, adequate intervention content, features meeting user needs*) are also the most common reasons for engaging with apps highlighting the need for the development of effective mental health applications. Users in both studies mentioned engaging with apps that improve their mental state, offer a variety of content, several customization options, and the ability to use them in different contexts and at any time. Thus, users value apps that are free or at an affordable price, offer an aesthetically pleasing interface, are easy to use, and can be used in a time-efficient manner. Our results are in line with previous studies that explored functionality and aspects that affect user experience with mental health apps (32,34–36).

Participants in both studies provided suggestions for improvement to increase engagement and sustained use. Users during the interviews often requested personalization of the experience by receiving content related to their needs based on their interaction with different app features, and the ability to customize app content. Similarly, the results of the user reviews analysis showed that users are dissatisfied with apps that provide content not relevant to their unique inputs and that lack customization options. Previous research has shown that personalizing the user experience and customizing app content are critical factors affecting user engagement with mental health apps (32,35).

Self-management apps often lack guidance on how to use an app and do not provide an explanation of the benefits of different app features. Interestingly, a few users in the interview study requested the integration of psychological data to capture physical signs and symptoms that occur before or during anxiety events. A growing body of research has started exploring users’ preferences in sharing their data for receiving tailored content (63,64), and potential opportunities for such technologies (65). In the next section, we highlight the main barriers to engagement with mental health apps for anxiety anxiety management identified in both studies, leading to implications for design.

### 7.2. Main barriers to engagement

#### 7.2.1. App classification

Users’ needs and expectations differ and apps are used in diverse contexts. Therefore, users should be informed about an app’s benefits and features both before downloading an app and during use. The description page in the app stores should specify the target audience of an app to increase user adoption. Developers should consider the demographics of potential users and specify the types of anxiety disorders that can be addressed through a specific app. Apps need to be classified in ways that are useful and meaningful to potential users by addressing problems that they have, skills that they need, or features that they’re interested in (66). The information available on the description page should inform potential users of these apps about the expected values and benefits of using the app. Additionally, privacy policies should provide clear information on which data is collected, how data is stored, if data is shared and with whom, and ensure data security to gain users’ trust. Several guidelines have been created for clinicians to assess such apps and evaluate those aspects (67–71). Future designs should ensure the provision of such information in the description pages to increase user adoption.

#### 7.2.2. Quality issues

Functionality issues such as bugs and crashes, and interface design issues such as poor UI, lack of app guidance, and difficulties in navigation restrain app use and prevent adoption and sustained engagement. Additionally, the apps restrict access to people with disabilities and are not necessarily compatible across different devices. These issues, identified within both studies, suggest a need for greater use of user-centered design methods during the development of apps, and following deployment. A key role for HCI professionals (including researchers) in the future is thus to ensure that issues of safety, usability, utility and user experience are addressed (72). Such technologies should be well engineered (hardware and software perform as intended), be safe and clinically effective, and should address user-centred requirements at different levels (being usable, useful, engaging, and fitting the context of use) (72). Future work could evaluate the coherence between the different app features by analyzing quantitative data regarding the number of positive and negative comments or based on the mean scores in the app store. In addition, future work on the prevalence of different barriers could draw on our analysis in the preparation of a codebook. Future research should consider end-users during different stages of an app’s implementation process and in different contexts of use.

#### 7.2.3. Costs

Costly in-app purchases and subscriptions can also be a barrier to sustained engagement with the apps. Studies show that free apps are more likely to be downloaded than paid apps (73). In addition, users complained about inadequate content in the free version of apps limiting users from realizing an apps’ potential to improve their mental health, and to make informed judgements regarding the value of paid versions.

#### 7.2.4. Personalization and customization

Current apps are not designed by taking into consideration diverse target audiences, user needs, and contexts of use. The delivery of different functionality features is not considering specific group characteristics that may impact an app’s adoption. Participation in an intervention may be influenced by variability in age, education level, symptom burden, cognitive capacity, sociocultural influences, and other differences.

Previous studies have explored the design of tailored mobile interfaces based on collected data from participants or based on participants’ preferences (74–76). Other studies have explored the design of mobile interfaces based on different user characteristics such as the level of literacy (77), and language barriers (78) or even the design of specific functionality types such as chatbots (79). A language-free app was developed without any text to guide refugee users through specific therapeutic techniques and can be accessed by multiple cultural groups irrespective of the language of origin (78). Future research could further explore universal concepts that can be applied in the design of MHapps. Users value options for content variety that can be customized based on their needs. Users should be allowed to customize different app features to meet their preferences and functionalities that be used in different contexts. Future studies should explore which customization options are valued for specific groups of people and for different app functionalities.

Similarly, apps should personalize an experience with data collected through the app. Research shows that personalizing app content is critical for improving engagement with mental health apps (27,32,35,41,80). We should consider ways to personalize an experience in different stages of a user journey. When the user journey involves mental health recovery, this personalization should take into consideration the nature of anxiety. For example, previous HCI work has examined how the pathology of bipolar disorder impacts on the design of mHealth interventions (81). Anxiety symptoms might increase or diminish when people may be more or less exposed to certain objects or situations, which impacts on the functionality which is most helpful at a given point in time. Future studies should explore effective ways to personalize the user experience with data collected during initial download and during app use. Such data can be collected based on collected answers from questionnaires, users’ app history and interaction with app features, users’ preferences for content delivery, and even through ubiquitous collection of data with the use of sensors, as in the case of ecological momentary interventions (82). Future studies should explore whether the personalization of content should be automated or configured by the users. Additionally, existing users will benefit from adaptive content delivered based on their time interacting with an app. Future research is needed to understand the stages through the user journey where content becomes repetitive and adapt it to sustain engagement.

#### 7.2.5. In-app support

Supporting users of such apps throughout their journey is important for sustaining engagement. Users requested guidance during their interaction, effective customer support to address their queries, and value in-app social features. Previous research has shown that the most effective apps in efficacy and effectiveness tend to be those with human support, likely through their ability to boost engagement (83). Users value the option to contact a therapist through an app, even though the technological limitations of such an implementation, such as the text-based nature of therapist-user interaction, can impact use negatively. Assessing and optimizing the digital therapeutic alliance holds the potential to make such tools more effective and improve adherence to their use (84). Additionally, research suggests that mental health apps for anxiety often lack the involvement of experts during their development (8,9,85). Therefore, future HCI research should explore ways to increase therapist involvement in the design process and explore ways to optimize therapeutic alliance through an app.

Users with mental health issues value sharing and communicating with others through an app in line with the results of a previous study (41). There is hence an opportunity for HCI research to explore how to effectively integrate such functionality while minimizing the risks of miscommunication or misinformation by other users.

### 7.3. Recommendations

The studies showed that there is a diversity in users’ needs and the context that apps are used. HCI designers should consider the user experience in different stages of the user journey. Apps should be developed by using user-centered design methods to ensure good functionality and accessibility for diverse target audiences and needs. Customer support should be available for all users. In addition, HCI researchers should design apps considering different group characteristics and preferences. App developers should classify apps’ benefits and value on the description page of the app stores to enhance the adoption of such apps. During their interaction with the apps, use guidance, customization of different app features, and personalization of the experience are essential factors to provoke user sustained engagement. Intervention content should be adapted based on users’ needs and interactions over time. This can be achieved by delivering new content at different time points of app use. Lastly, in-app therapists and social support can enhance regular use and improve app adoption. Usage of these features can be improved by supporting video therapy sessions and by monitoring discussion groups. Apps should offer valuable intervention content in the free version and offer alternative payment plans to satisfy the diverse user needs. Applying human-centered design in different stages of an app’s design cycle is essential to address the identified barriers in our study and improve users’ interactions with app features to support long-term use.

## 8. Limitations

Both studies made use of feedback from users of mobile apps targeting anxiety disorders. Potential limitations regarding the generalizability of the findings are taken into account when interpreting users’ feedback. Our design recommendations are based on users’ needs and expectations from mobile apps for anxiety management. However, this work does not incorporate the perspectives of clinicians which may be complementary in terms of understanding opportunities for self-management. Our participants’ experiences may not be representative of wider audiences since demographics and app use characteristics vary in our study. The apps used in the first study are not representative of the apps available in the app stores due to the selection criteria of the study (CBT apps that present some evidence for effectiveness for anxiety management) and the fact that most apps in the app store do not have evidence supporting their effectiveness (9). Thus, understanding the perspectives of a different set of stakeholders or a different set of apps might reveal different barriers, facilitators, or design considerations. Additionally, we did not take any clinical measures to determine if participants in both studies met the criteria for anxiety disorders or to understand the intensity of their feelings. Overall, our approach provided data on experience across a number of different apps used in different settings in daily life. User reviews analysis of individual apps provided a large volume of evaluation data, whereas the interview study provided more detailed insight into in-the-wild usage of such apps.

## 9. Conclusions

Understanding users’ perspectives on the burden and engagement with mobile apps for anxiety are important to design apps for sustained use. We conducted two qualitative studies based on user reviews from app marketplaces, and interviews with users of such apps. This paper extends the existing literature by highlighting and distinguishing barriers preventing adoption and sustained use, and by exploring users’ experiences with apps targeted for anxiety management. This paper provides a focused perspective on users’ attitudes and expectations towards mental health apps for anxiety, contributing to an important topic at the intersection of HCI and mental health. We have revealed negative and positive aspects affecting the experience with available anxiety apps, as well as a range of unmet needs, and suggested improvements. Finally, we provide design implications to designers and developers of anxiety mental health apps to improve user adoption and sustained use. Addressing these factors can help realise the potential of mental health apps to support self-management of anxiety management.

## Data Availability

The original contributions presented in the study are included in the article/Supplementary Material, further inquiries can be directed to the corresponding author/s.

## Footnotes

^1^If participants experience discomfort during the interviews, they were advised to let the facilitator know, and take a break. If a participant experiences significant distress, the facilitator would advise them to withdraw from the study and seek help. They will refer them to the local emergency number provided at the beginning of the interview session. In addition, the facilitator also would provide the respondents with online resources on mental wellbeing for the cases when they might have any concerns or discomfort regarding the topics covered in this study.
